# Reactivation of Hepatitis B Virus in Patients with Multiple Myeloma

**DOI:** 10.3390/cancers11111819

**Published:** 2019-11-19

**Authors:** Yutaka Tsukune, Makoto Sasaki, Norio Komatsu

**Affiliations:** Department of Hematology, Juntendo University School of Medicine, 2-1-1 Hongo, Bunkyo-Ku, Tokyo 113-8421, Japan

**Keywords:** hepatitis B virus, reactivation, multiple myeloma, novel agents, autologous stem cell transplantation

## Abstract

Reactivation of hepatitis B virus (HBV) is a well-known complication in patients with hematological malignancies during or after cytotoxic chemotherapy. If the initiation of antiviral therapy is delayed in patients with HBV reactivation, these patients can develop severe hepatitis and may die of fulminant hepatitis. The preventive strategy for HBV reactivation in patients with malignant lymphoma has already been established based on some prospective studies. As there was an increased number of novel agents being approved for the treatment of multiple myeloma (MM), the number of reported cases of HBV reactivation among MM patients has gradually increased. We conducted a Japanese nationwide retrospective study and revealed that HBV reactivation in MM patients is not rare and that autologous stem cell transplantation is a significant risk factor. In this study, around 20% of all patients with HBV reactivation developed HBV reactivation after 2 years from the initiation of therapy, unlike malignant lymphoma. This might be due to the fact that almost all of the patients received chemotherapy for a long duration. Therefore, a new strategy for the prevention of HBV reactivation in MM patients is required.

## 1. Introduction

An estimated 2 billion people worldwide have evidence of either current or past hepatitis B virus (HBV) infection, including 257 million people with chronic HBV infection (HBV carriers), who are defined as testing positive for hepatitis B surface antigen (HBsAg) [1,2]. Specifically, the prevalence of past HBV infection is particularly high in Asia (Japan: 23.2%, China: 20.1%, Singapore: 34.3%, Hong Kong: 44.2–62.0%) [3]. HBV reactivation is a well-known complication in patients with hematological malignancies during or after cytotoxic chemotherapy. HBV reactivation is caused by regrowth of HBV in the body, and it can induce severe flares of hepatitis, which can in turn lead to fatal fulminant hepatitis [4]. HBV reactivation has mainly been observed in HBV carriers who were receiving cancer chemotherapy [4,5]. On the other hand, HBV reactivation is also known to occur in patients with resolved HBV infection, with resolution being defined as testing negative for HBsAg but positive for antibodies against hepatitis B core antigen (anti-HBc) and/or antibodies against hepatitis B surface antigen (anti-HBs) [6]. HBV reactivation in these patients used to occur mainly after hematopoietic stem cell transplantation or solid organ transplantation. However, after the approval of rituximab, HBV reactivation in non-Hodgkin lymphoma patients has increased. Rituximab is a humanized monoclonal antibody against CD20, which is expressed on the surface of B lymphocytes and is used to treat B-cell non-Hodgkin lymphoma. Moreover, there have been subsequent reports of HBV reactivation in cases of immune-chemotherapy or immunosuppressive therapy for other malignancies or autoimmune disorders [7,8,9,10]. These patients included not only HBV carriers but also those with resolved HBV infection [11,12,13]. There have been several prospective studies on HBV reactivation among patients with malignant lymphoma in Asia [14,15,16,17,18]. The cumulative incidence rate of HBV reactivation in patients with resolved HBV infection who were treated with rituximab and steroid-containing regimens was reported to be 8.3% in a 1.5-year study [18] and 41.5% in a 2-years study [17]. Of these, some patients developed HBV reactivation-related hepatitis and liver failure. Some risk factors and preventive strategies for HBV reactivation-related hepatitis have been reaching a consensus. Recently, HBV reactivation-related hepatitis and liver failure have been recognized as an important complication of cancer chemotherapy and immunosuppressive therapy.

Multiple myeloma (MM) is a neoplastic proliferation of plasma cells that are derived from B cells presenting various clinical conditions, such as anemia, renal dysfunction, osteolytic lesion, and hypercalcemia. Before the turn of the millennium, melphalan-prednisolone (MP) was the standard therapy for older patients, and high dose melphalan with autologous stem cell transplantation (ASCT) was the standard therapy for younger patients. However, the median overall survival (OS) was 38.9 months for the 1990–2000 cohort among Japanese MM patients [19], and accordingly, MM was recognized as a poor prognostic disease. After the turn of the millennium, the appearance of new treatment agents, including proteasome inhibitors, immunomodulatory drugs (IMiDs), and monoclonal antibodies, have significantly improved prognoses [20,21]. Among Japanese MM patients, the median OS was 60.6 months in the 2001–2012 cohort [19]. Before the use of these novel agents, HBV reactivation hardly occurred in patients who underwent ASCT. However, since then, cases of HBV reactivation have begun to be reported more commonly. Our previous retrospective study showed that HBV reactivation in MM patients was not rare and was significantly higher among patients who underwent ASCT than among those who did not undergo ASCT [22]. Therefore, we review HBV reactivation and its preventive strategy, based on a nationwide retrospective analysis and on other reports regarding MM patients.

## 2. HBV Reactivation

HBV has a very long-lasting form of the virus called covalently closed circular DNA (cccDNA). HBV persists in hepatocytes in patients with a history of transient HBV infection. Cellular and humoral immune responses commonly suppress viral replication. However, impairment of the host immune system due to chemotherapy or immunosuppressive therapy can result in active replication of HBV and a high viral load of HBV in the blood. Then immune reconstitution occurs, with T lymphocytes that recognize HBV attacking the liver, trying to clear HBV from the liver and leading to flares of hepatitis [23]. The incidence of HBV reactivation has been increasing since the approval of rituximab, suggesting that the function of B lymphocytes may well be more important. One of the functions of B lymphocytes is the production of neutralizing antibodies. It was reported that the presence of neutralizing antibodies contributes to HBV clearance [24]. They also speculated that B lymphocytes may have additional functions in suppressing HBV replication.

HBV reactivation was defined at a conference of the American Association for the Study of Liver Disease in 2013 [25]. The definition of HBV reactivation depends on the baseline virologic profile. HBV reactivation in HBV carriers is defined as a ≥2 log_10_ increase in HBV DNA levels from baseline levels, the detection of HBV DNA with level ≥ 100 IU/mL in HBV carriers with undetectable HBV DNA at baseline, or the detection of HBV DNA with level ≥100,000 IU/mL in HBV carriers with no baseline HBV DNA data [25]. HBV reactivation in patients with resolved HBV infection is defined as the reappearance of HBsAg or the appearance of HBV DNA in the absence of HBsAg [25]. We classified the severity of HBV reactivation into five groups (Table 1) [24,26]. Although the vast majority of cases of HBV reactivation in MM patients correspond to virological changes that are not clinically significant, HBV reactivation can lead to death or necessitate the interruption of treatment.

The risk factors of HBV reactivation fall into three categories: (1) virological factors, (2) treatment factors, and (3) host factors [24]. First, the virological factors related to HBV reactivation include a high viral load at baseline, hepatitis B e antigen (HBeAg) positivity, and HBsAg positivity. A relationship between HBV genotype or mutations and HBV reactivation was reported recently [27]. Blood samples from 36 patients (17 with HBV reactivation and 19 with acute hepatitis) were analyzed using next-generation sequencing. The number of patients with genotype Bj was significantly higher in the HBV reactivation group than in the acute hepatitis group. The prevalence of the S3N amino acid substitution in the envelope protein, and *G1896A* and *G1899A* mutations in the pre-core region, was also significantly higher in the HBV reactivation group than in the acute hepatitis group.

HBV reactivation is affected by the virological state of HBV and the condition of the host’s immune system. Patients with HBV infection may be classified into five phases: (1) immune-tolerant, (2) immune-active HBeAg-positive carrier, (3) inactive carrier, (4) HBeAg-negative chronic hepatitis, and (5) resolved infection [24,26,28,29,30]. This classification is based on patients’ levels of alanine aminotransferase (ALT), HBV serological markers (HBsAg, anti-HBs, anti-HBc, HBeAg, antibodies against the hepatitis B e antigen [anti-HBe], and viral load). Immune-active HBeAg-positive carriers and patients with HBeAg-negative chronic hepatitis are characterized by high viral loads (≥2000 IU/mL), and ALT elevation [26,29]. An inactive carrier is also known as immune control and defined as testing positive for anti-HBe, having undetectable or low HBV DNA levels (<2000 IU/mL) and normal ALT levels [26,29]. Resolved infection is characterized by testing negative for HBsAg but positive for anti-HBc and/or anti-HBs with undetectable levels of HBV DNA [26,29]. The risk of HBV reactivation is higher for HBsAg-positive patients (immune-active HBeAg-positive carriers and patients with HBeAg-negative chronic hepatitis) than for HBsAg-negative patients (resolved infection). In a study of patients with B-cell non-Hodgkin lymphoma, the incidence rate for HBV reactivation in HBsAg-positive patients was higher than that in patients with resolved infection (20–50% vs. 1.0–2.7% in patients on conventional chemotherapy, >50% vs. 14–20% in patients who have had hematopoietic stem cell transplantation [HSCT], and 80% vs. 12.2–23.3% in patients on rituximab-containing regimens) [3]. Among HBsAg- positive patients, consultation with a hepatologist should be required to assist with decisions regarding the use of chemotherapy, immunosuppressive therapy, and antiviral therapy.

Second, the treatment factors can be divided into high risk, moderate risk, and low risk [30,31,32]. The several risk factors of treatment of HBV reactivation are solid organ or hematopoietic stem cell transplantation and the administration of rituximab, antitumor necrosis factor-α (TNF-α) agents, steroids, and anthracycline [13,15,33,34,35,36,37]. The risk classification of treatments and drugs in MM is summarized in Table 2. Histone deacetylase inhibitors are classified into moderate risk groups [31,32]. However, we classified panobinostat usually combined with bortezomib for MM patients into the unclassifiable group, because there were no reports on panobinostat and HBV reactivation.

Third, the host factors, such as being male, being of older age, having elevated ALT levels, having hematopoietic malignancies, and having breast cancer, have also been reported [24,30,33,36,37,38].

A retrospective study by the Asian Lymphoma Study Group defined HBV reactivation as the reappearance of HBsAg, in B-cell lymphoma patients who received rituximab-containing regimens [39]. In this study, HBV reactivation occurred in 19 of 261 patients, including four patients who died because of HBV reactivation-related hepatic failure despite the administration of antiviral agents (specifically, lamivudine [LAM] or entecavir [ETV]). In the process of HBV reactivation, a Chinese retrospective analysis presented crucial information that the median time from the elevation in serum HBV DNA levels to HBsAg seroreversion or hepatitis onset was 10 weeks (range, 8–12 weeks) and 18.5 weeks (range, 12–28 weeks), respectively [12]. Based on these facts, the diagnosis of HBV reactivation and administration of an antiviral agent need to be performed earlier than the reappearance of HBsAg.

HBV reactivation has been analyzed in four large-scale prospective studies of patients with malignant lymphoma and resolved HBV infection who received chemotherapy with or without rituximab [16,17,18,40] (Table 3). The Taiwan Cooperative Oncology Group conducted a prospective study of 150 HBsAg-negative lymphoma patients receiving rituximab-CHOP (cyclophosphamide, doxorubicin, vincristine, prednisolone)-based chemotherapy [16]. They defined HBV reactivation as HBV DNA levels ≥10-fold above baseline levels and HBV reactivation-related hepatitis flares as a ≥3-fold increase in serum ALT level that exceeded 100 IU/L. They started ETV administration at the point of HBV reactivation. However, 17 of 150 patients experienced HBV reactivation, including 10 who developed HBV reactivation-related hepatitis flares. Thus, the study showed that the administration of antiviral agents at a point determined by HBV DNA levels might also be too late.

A prospective study of B-cell non-Hodgkin lymphoma patients with resolved HBV infection treated with rituximab-containing regimens was performed in Hong Kong [17]. This study was conducted using a quantitative test for HBV DNA, in which a real-time polymerase chain reaction (RT-PCR) assay was performed every 4 weeks. Reactivation was determined to be the point at which any detectable levels of HBV DNA were found, and ETV was started at this point. In this study, HBV reactivation was observed in 19 of 63 cases (30.2%), and none of the cases developed hepatitis.

A prospective study in Japan also reported a similar protocol for patients with B-cell non-Hodgkin lymphoma and resolved HBV infection who received chemotherapy including rituximab and steroids [18]. HBV reactivation was observed in 22 of 269 patients (8.2%), and none of these patients developed hepatitis. HBV DNA genotypes and mutations were evaluated in these 22 patients, and no patients with ETV resistance were observed. On the other hand, mutation of the pre-core region (*G1896A*), which is detected at a high rate in patients with fulminant hepatic failure, and mutation in the core promoter region (*A1762T/G1764A*), which is determined to accelerate virus growth radically, were observed in six cases, and HBV DNA levels increased more rapidly in these six patients than in others. However, in these patients, the onset of hepatitis could be prevented by ETV administration.

Recently, a Chinese group reported a prospective randomized controlled study for patients with malignant lymphoma and resolved HBV infection, to evaluate the efficacy and safety of ETV prophylaxis [40]. Patients were randomly assigned in a 1:1 ratio to either a prophylactic ETV group or a preemptive therapy group. HBV reactivation was defined as a reappearance of HBsAg and HBV DNA detection. In the prophylactic ETV group, antiviral administration was started from the initiation of chemotherapy to at least 6 months after the final dose of chemotherapy. In the preemptive therapy group, antiviral therapy was initiated as soon as patients were diagnosed as having HBV reactivation. The primary endpoints were the incidence rates of HBV reactivation and HBV reactivation-related hepatitis. The incidence rates of HBV reactivation and HBV reactivation-related hepatitis were 0% (0/95) and 0% (0/95), respectively, in the prophylaxis group and 3.2% (3/95) and 1.1% (1/95), respectively, in the preemptive therapy group. There was no significant difference between the two therapy groups. One patient developed HBV reactivation-related hepatitis in the preemptive therapy group, but the authors did not discuss the reason for the onset of hepatitis. We thus deduced that the initiation of the antiviral agent at the time of HBsAg reappearance could be too late.

Based on these data, both routine monitoring of HBV DNA levels and starting administration of an antiviral agent at the point of detectable HBV DNA (preemptive therapy) have been recommended in some guidelines [41,42]. However, other guidelines and a recent review article stated that prophylactic administration of antiviral drugs is recommended for high-risk patients who receive regimens containing B-cell depleting agents or undergo HSCT [28,30,43,44,45]. 

Recent global studies have evaluated the incidence of HBV reactivation in B-cell non-Hodgkin lymphoma patients with resolved HBV infection who received immunochemotherapy containing B-cell depleting agents (obinutuzumab or rituximab) in phase III GOYA and GALLIUM studies [46]. Among 326 patients identified as being at high risk of HBV reactivation, 27 (8.2%) experienced HBV reactivation. In the 232 patients without prophylactic nucleos(t)ide analog (NA) treatment, 25 (10.8%) experienced HBV reactivation; these 25 patients received preemptive NA therapy, and none of them developed HBV reactivation-related hepatitis. In the 94 patients who received prophylactic NA treatment, two patients who received LAM (2.1%) experienced HBV reactivation. Based on these studies [17,18,46], we concluded that regular monitoring of HBV DNA and preemptive NA therapy is the most suitable for preventing HBV reactivation-related hepatitis among B-cell non-Hodgkin lymphoma patients.

## 3. HBV Reactivation in MM Patients

Retrospective reports summarizing HBV reactivation in MM patients, including case reports, included a total of 125 cases [8,22,47,48,49,50,51,52,53,54,55,56,57,58,59,60,61,62,63,64,65,66,67,68] (Table 4 and Table 5). All 16 cases with HBV reactivation through 2010 underwent HSCT [56,57,58,59,60,61]. After 2010, the number of cases of HBV reactivation in patients receiving novel agents without HSCT increased. Only one case was reported after 2010 in which a patient received conventional MP therapy alone [8].

HBV reactivation has been reported in two relatively large-scale retrospective studies of patients with MM and resolved HBV infection from Korea and Japan [22,62]. A Korean study reported about 628 MM cases in a single institution [62], and the researchers defined HBV reactivation as the reappearance of HBsAg and analyzed 230 cases (36.6%) with resolved HBV infection. During a median follow-up period of 2.4 years, HBV reactivation was observed in 12 cases (5.2%). The cumulative incidence rate of HBV reactivation at 2 and 5 years was 5% and 8%, respectively. ASCT was performed in 127 patients, including all 12 with HBV reactivation. The researchers revealed that ASCT and anti-HBs negativity were risk factors for HBV reactivation. In addition, the researchers defined biochemical flare as the elevation in serum ALT levels (≥80 U/L), which was observed in eight of the 12 patients.

Our first study reported that, among 641 Japanese MM patients, one of eight (12.5%) HBV carriers developed hepatitis, and nine of 99 (9.1%) patients with resolved HBV infection experienced HBV reactivation [64]. Furthermore, the cumulative incidences of HBV reactivation at 2 and 5 years were 8% and 14%, respectively. Univariate analysis identified elevated serum albumin level as the only risk factor for HBV reactivation. This association could not be reasonably explained. We concluded that HBV reactivation was not rare, although risk factors and optimal preventive strategies for HBV reactivation were not identified. Therefore, we conducted a nationwide retrospective study aimed at evaluating the actual incidence and risk factors of HBV reactivation.

Our Japanese nationwide study collected data of 5,078 MM patients from 76 Japanese hospitals [22]. All patients were either treated with novel agents (bortezomib, thalidomide, lenalidomide, pomalidomide, panobinostat, carfilzomib, elotuzumab, and ixazomib) or underwent ASCT. Of these patients, 760 (15.0%) exhibited resolved HBV infection. HBV reactivation was defined as the detection of HBV DNA in the peripheral blood during or after treatment. During the median follow-up period of 101 (1–540) weeks, HBV reactivation was observed in 58 cases (7.6%). The cumulative incidence rates of HBV reactivation at 2 and 5 years were 7.9% and 14.1%, respectively. The cumulative incidence of HBV reactivation was higher in patients who underwent ASCT (16.0% at 2 years; 30.6% at 5 years) than in those who did not (4.4% at 2 years; 4.8% at 5 years). Ten of 58 patients (17.2%) with HBV reactivation developed hepatitis, and one of these patients died of fulminant hepatitis despite the administration of the antiviral agent. These 10 patients were not regularly monitored for HBV DNA levels; instead, HBV reactivation was diagnosed after an elevation in ALT levels was observed. The other 48 patients were subjected to regular HBV DNA monitoring and preemptive antiviral therapy as per Japanese guidelines [23], and none developed HBV-reactivation-related hepatitis. In the univariate analysis, a high incidence of HBV reactivation was observed in groups with elevated serum albumin levels, of younger age, with decreased gamma-glutamyl transpeptidase levels, and that underwent ASCT. Multivariate analysis revealed that ASCT was a strong risk factor of HBV reactivation. In contrast, lenalidomide treatment was associated with a low prevalence of HBV reactivation.

Thus, we identified a general trend in these Korean and Japanese studies showing that HBV reactivation is not rare among MM patients, especially among patients who underwent ASCT.

## 4. The Prophylactic Strategy for HBV Reactivation in MM Patients

There are no prospective studies or established guidelines on HBV reactivation in MM patients. In the previously described two retrospective studies among MM patients, the cumulative incidence rate of HBV reactivation was higher and the rate of biochemical flare was lower in the Japanese study than in the Korean study [22,62]. The difference in these rates may be attributed to the different definitions used for HBV reactivation in each study. Similarly, from the reports on malignant lymphoma, we identified that a diagnosis of HBV reactivation at the point of HBsAg seroconversion was too late for the prevention of HBV reactivation-related hepatitis. Meanwhile, the prospective study of Japanese patients with malignant lymphoma showed that management including regular HBV DNA monitoring and/or preemptive antiviral therapy was practical [18], and none of the patients in our study who were treated in the same way developed either HBV reactivation-related hepatitis or liver failure [22]. We suggest that this strategy of HBV DNA monitoring and preemptive therapy is the most reasonable and practical considering the present understanding and analytical capabilities.

The antiviral agents LAM and ETV have been widely used for the prevention of HBV reactivation-related hepatitis. Among patients with B-cell non-Hodgkin lymphoma and HBV carriers, a randomized controlled study showed a significantly lower incidence of HBV reactivation and HBV reactivation-related hepatitis in the study’s ETV prophylactic group than in its LAM prophylactic group [69]. The authors suggested that the reason for the better outcome of ETV prophylactic group was that ETV had potent antiviral activity and a high genetic barrier to resistance. Other studies showed that long-term administration of LAM was significantly associated with drug resistance [46,70,71]. ETV was used to address HBV reactivation in a Japanese prospective study of malignant lymphoma, in which there were no cases that showed resistance to ETV [18]. The duration of treatment in MM patients is usually longer than that in lymphoma patients. Therefore, although we currently recommend ETV for the prophylaxis of HBV reactivation-related hepatitis, we also propose that a consultation with a hepatologist should be required at the time of HBV reactivation. 

Some recent guidelines recommend other NAs, such as tenofovir disoproxil fumarate (TDF) and tenofovir alafenamide (TAF), for the prevention of HBV reactivation [28,72]. In recent studies, TDF completely prevented the onset of HBV reactivation and the development of HBV reactivation-related hepatitis in malignant lymphoma patients [73,74]. In addition, TDF has no reported occurrences of resistant mutation after 6 years of treatment [75]. 

However, it is necessary to pay attention to the administration of NAs because renal dysfunction and reduction in bone mineral density are known to be adverse effects of these agents and because the effects correspond to organ damage caused by MM [76,77]. It was reported that less than 1% of patients treated with ETV had elevated serum creatinine [78]. TDF may result in mild proximal tubular dysfunction, and renal toxicity tends to increase with cumulative exposure. There was a report on the 7-year efficacy and safety of TDF treatment in 585 chronic hepatitis B patients, of whom only 10 (1.7%) experienced elevation in serum creatinine levels ≥0.5 mg/dL at baseline [79]. Compared with TDF, TAF is said to cause less renal toxicity. However, ETV and TDF require dose adjustments based on creatinine clearance values. Thus, we need to carefully observe renal function when ETV and TDF are used for MM patients. Bone events following NA treatment did not increase significantly, except for hip fracture in a large cohort study [80]. However, MM patients with osteolytic lesions or bone plasmacytoma need to be closely monitored by means of bone density testing.

In malignant lymphoma studies, the median duration from the initiation of chemotherapy to the occurrence of HBV reactivation was less than 6 months [16,17,18] (Table 2). The late onset of HBV reactivation (“late” being defined as occurring between 6 and 12 months after completion of therapy) for rituximab-containing immunochemotherapy rarely occurs in B-cell non-Hodgkin lymphoma patients [46,81,82]. Conversely, 38 patients who underwent ASCT experienced HBV reactivation (median, 55 weeks; range, 10–250 weeks), and six patients of those had HBV reactivation more than 2 years after transplantation in our study [22]. Most of these patients were receiving chemotherapy owing to the recurrence of MM. However, one case developed HBV reactivation after ASCT despite complete remission and more than 4 years after cessation of chemotherapy. Moreover, in our study, while most of the 20 cases of HBV reactivation without ASCT occurred within 2 years since treatment initiation, one of them occurred after more than 6 years. Unlike malignant lymphoma, most MM patients receive treatment for a long time because MM is an incurable disease. A new strategy, with maintenance therapy and salvage therapy including novel agents, has been improving the prognosis of MM patients [83,84]. Consequentially, the use of novel agents has led to a more prolonged term treatment for MM patients. The regular monitoring of HBV DNA is necessary for at least 12 months after treatment completion in Japanese guidelines [23]. However, late HBV reactivation has occurred more frequently in MM patients than in lymphoma patients, as previously demonstrated by the over 6 year interval before reactivation was observed in our patient. HBV DNA monitoring for MM patients, especially those who underwent ASCT, should probably be required for quite a longer time and more carefully than that for lymphoma patients, even after treatment is discontinued. 

According to the Japanese guidelines, cessation of antiviral therapy can be considered if the following three criteria are met: (1) antiviral therapy has been continued for at least 12 months after the completion of chemotherapy or immunosuppressive therapy; (2) ALT levels have been normalized during this period (except, for example, cases with fatty liver and drug-induced liver dysfunction); and (3) HBV DNA levels are continuously below detectable limits during this period (and, ideally, HBsAg and hepatitis B core-related antigens are also continuously negative) [23]. Both a recent Australian consensus statement and other guidelines recommend that patients with resolved HBV infection who previously received B-cell-depleting agents or underwent HSCT require prophylactic NA therapy for 12–24 months after treatment completion and continued HBV DNA monitoring for at least 12 months after prophylaxis withdrawal [28,45,85]. The same consensus statement recommended that patients with other chemotherapies continue prophylactic NA therapy for 6–12 months. However, this statement is not enough for MM patients. The optimal period of prophylactic NA therapy for MM patients may need longer durations.

The main merit of preemptive therapy is the avoidance of unnecessary administration of antiviral agents. In our study, only 5% of patients who were ineligible for transplantation needed antiviral therapy [22]. Antiviral agents may be used as prophylaxis in patients who do not require antiviral therapy. In the previously described Chinese study [40], the incidence rates of HBV reactivation and HBV reactivation-related hepatitis in the prophylactic antiviral therapy group were not significantly higher than those in the preemptive therapy group. This study also assessed cost-effectiveness. The base-case annual costs were $2,536 per patient in the prophylactic ETV group and $1,039 in the control group. The cost of the prophylactic ETV group was higher than that in the control group ($125 per month per patient). Continuous prophylaxis using antivirals during and after chemotherapy for MM is not economical, because the duration of treatment is usually longer in MM patients than in lymphoma patients. The main drawback of preemptive therapy is that patients are provided with preemptive therapy may develop severe hepatitis owing to a delay in the diagnosis of HBV reactivation. HBV reactivation may not be detected in a timely fashion in routine practice rather than in the setting of a clinical trial. In fact, 10 patients received a delayed diagnosis of HBV reactivation and experienced hepatitis in our study. Moreover, HBV DNA testing is very expensive, and it takes a long time to obtain the result in some regions.

On the other hand, the advantage of prophylactic therapy is the suppression of HBV replication and the decrease in the incidence rate of HBV reactivation. The disadvantages of prophylactic therapy are the appearance of resistant mutations and adverse events caused by antiviral agents. Further research is needed on how to resolve these clinical issues.

In resource-limited settings, access to diagnostic tests and the availability of treatment are limited and are not affordable. In these countries, a specialized strategy to manage HBV reactivation in MM patients is needed.

## 5. Effect of Novel Agents on HBV Reactivation

The effects of bortezomib include the suppression of T lymphocyte proliferation and a decrease and hypofunction of both natural killer cells and CD8-positive T cells [86]. Treatment with bortezomib may be a major risk factor for the reactivation of herpes simplex virus and varicella-zoster virus (VZV), and thus, the prophylactic coadministration of antiviral agents against VZV is recommended [86]. Similarly, bortezomib might also be considered a possible risk factor for HBV reactivation. A recent report revealed that c-Abl, which regulates cell growth and survival, promotes cellin-RING ligase 4 mediated ubiquitination of HBV polymerase and further suppresses HBV replication [87]. Bortezomib blocks the ubiquitination of HBV polymerase by inhibiting c-Abl kinase activity in vitro and in vivo, and this mechanism may contribute to increasing cases with HBV reactivation.

The first HBV reactivation case treated with bortezomib was reported in Japan [48], and reports on HBV reactivation cases associated with bortezomib have subsequently increased. Li et al. retrospectively reported about 139 Chinese MM patients who underwent bortezomib-containing chemotherapy [63]. In this report, HBV reactivation occurred in 7.7% of patients who were HBV carriers and who were treated with bortezomib-containing therapy and in 1.3% of patients treated with conventional chemotherapy, without ASCT and novel agents. One case with resolved HBV infection experienced HBV reactivation during chemotherapy including bortezomib. Meanwhile, some patients who received carfilzomib-containing regimens also experienced HBV reactivation [68]. HBV reactivation owing to ixazomib has not been reported as of now. Proteasome inhibitors have been recognized as a risk factor of HBV reactivation [31].

There have only been a few reports on the incidence of HBV reactivation among MM patients who were treated with lenalidomide- or pomalidomide-based regimens [54,88]. Our nationwide study revealed that lenalidomide significantly reduced HBV reactivation [22]. Of 58 patients who exhibited HBV reactivation, only one patient did not undergo ASCT and was not administered the novel agents without lenalidomide.

Lenalidomide is classified as an IMiD that targets cereblon (CRBN) to induce antitumor activity and immunomodulatory effects through the proliferation of immune effector cells such as T cells, natural killer cells, and dendritic cells. In the RNA-induced silencing complex, the argonaute 2 (AGO2) protein is the only member with catalytic activity and fulfills a central role, thereby regulating small-RNA-guided gene-splicing processes [89]. Xu et al. showed that AGO2 bound to CRBN and was negatively regulated by CRBN in MM cells [90]. In this study, lenalidomide significantly increased the expression of CRBN in myeloma cell lines. Conversely, lenalidomide decreased the levels of AGO2 and microRNAs. It was demonstrated that knocking down AGO2 induced a decrease in the levels of HBsAg and HBV DNA in cell lines transfected with a plasmid of HBV components [91]. Altogether, we speculated at the time that lenalidomide might inhibit HBV proliferation by decreasing AGO2 levels in MM patients [22]. Recently, The other group revealed that lenalidomide significantly enhanced interferon-α production by human plasmacytoid dendritic cells [92]. Interferon-α (currently pegylated interferon-α) is one of the main treatment options for active HBV hepatitis patients. This result suggests that immunomodulatory effects owing to lenalidomide may inhibit HBV proliferation in MM patients.

In addition, we can use monoclonal antibodies (e.g., elotuzumab and daratumumab) for MM patients. Currently, there are no reports on HBV reactivation owing to monoclonal antibodies. The European Society of Clinical Microbiology and Infectious Diseases Study Group for Infections in Compromised Hosts recently reported that daratumumab (with corticosteroid) might increase the incidence of HBV reactivation [93]; however, the mechanism for this is unknown. What is known is that daratumumab depletes CD38 positive regulatory T and B cells and myeloid-derived suppressor cells and increases cytotoxic T cell number and activation [94]. These cytotoxic T cells may attack HBV-infected hepatocytes, leading to HBV reactivation-related hepatitis.

## 6. Conclusions

A recent Japanese nationwide study revealed that among 31 patients with resolved HBV infection who developed HBV reactivation-related acute liver failure or late-onset hepatic failure owing to chemotherapy and/or immunosuppressive therapy between 2010 and 2015, 27 (87.1%) patients died [95]. HBV reactivation can lead to fatal fulminant hepatitis, and the incidence rate of HBV reactivation in MM patients has been increasing. 

In our study, no patients who were subjected to regular HBV DNA monitoring and preemptive antiviral therapy as per Japanese guidelines developed HBV reactivation-related hepatitis. We conclude that regular monitoring of HBV DNA and preemptive NA therapy is the most reasonable and practical for preventing HBV reactivation-related hepatitis among MM patients. However, the development of novel agents has dramatically improved the prognosis for MM. It is necessary to be aware that HBV reactivation can occur at any treatment phase and that regular monitoring of HBV DNA levels is required on a long-term basis. In addition, there remain some unresolved clinical variables regarding HBV reactivation in MM patients, such as the administration period for antiviral agents and the appropriate methods for HBV monitoring. Further large-scale prospective studies should aim to identify risk factors associated with novel agents more clearly and establish a new strategy to prevent HBV reactivation in MM patients.

## Figures and Tables

**Table 1:** Phases of HBV reactivation [24,26].

**Virological Changes**	
HBsAg negative to HBsAg positive	There is little clinical significance as it would not affect the care of myeloma treatment.
HBV DNA rising by 10-fold	Viral replication increases gradually. However, serum ALT and AST levels are normal and patients are asymptomatic. Increase in HBV DNA by 10-fold has little effect on chemotherapy.
**Clinically significances**	
HBV reactivation with hepatitis	ALT or AST levels become abnormal but not so high (2–10 times the upper limit of normal or baseline levels). This is important clinically as it might affect myeloma care.
Severe hepatitis without liver failure	ALT or AST levels become >10 times the upper limit of normal or baseline levels. PT-INR remains normal. This is important as it might lead to liver failure despite treatment with HBV antiviral therapy.
**Fulminant hepatitis/death**	
Severe hepatitis leading to liver failure	Liver failure is defined as (1) elevation in serum bilirubin level (>2 mg/dL) and prolongation of prothrombin time (PT-INR >1.3), (2) ascites or (3) encephalopathy. This is important as it can lead to death despite antiviral therapy.

Abbreviations: ALT, alanine aminotransferase; AST, aspartate aminotransferase; PT-INR, prothrombin time international normalized ratio.

**Table 2:** Risk classifications of treatments and drugs resulting in multiple myeloma.

Risk and Prevalence of HBV Reactivation	Treatments and Drugs
High risk (>10%)	Hematopoietic stem cell transplantation (autologous stem cell transplantation in almost cases)
Moderate risk (1–10%)	Proteasome inhibitors, such as bortezomib Anthracycline derivatives Moderate (prednisolone 10–20 mg daily or equivalent) or high-dose (prednisolone >20 mg daily or equivalent) corticosteroids daily for >4 weeks
Low risk (<1%)	IMiDs, such as thalidomide, lenalidomide and pomalidomide Alkylating agents Low-dose (prednisolone <10 mg or equivalent) corticosteroids for >4 weeks Any dose of oral corticosteroids daily for <1 week
Unclassifiable	Monoclonal antibodies such as daratumumab and elotuzumab Histone deacetylase inhibitor, such as panobinostat *

* In some reviews, histone deacetylase inhibitors are classified into the moderate risk group. However, we classified panobinostat into the “unclassifiable group”, because there are no reports that panobinostat induced HBV reactivation. Abbreviations: IMiDs, immunomodulatory drugs.

**Table 3:** Prospective studies on HBV reactivation in malignant lymphoma patients.

Study	Taiwan (2014) [16]	Hong Kong (2014) [17]	Japan (2015) [18]	China (2019) [36]
Number of patients	150	70	269	190 (prophylactic group: 95, preemptive group: 95)
Chemotherapy	R-CHOP	R containing regimens	R-steroid-containing regimens	Various regimens (R-containing regimens: 74.7%)
Definition of HBV reactivation	HBV DNA level; greater than 10-fold increase	HBV DNA level >10 IU/mL (1.7 log copies/mL)	HBV DNA level >11 IU/mL (1.8 log copies/mL)	Reappearance of HBsAg and HBV DNA
HBV DNA level; cut-off	3.0 log copies/mL	10 IU/mL (1.7 log copies/mL)	11 IU/mL (1.9 log copies/mL)	50 IU/mL
Duration from initiation of chemotherapy to HBV reactivation, median (range)	5.3 months (0.8–14.3)	6.0 months (1.0–25.0)	3.2 months (1.0–16.3)	6.0 months (2.0–7.0)
HBV reactivation	11.3% (17 of 150)	30.2% (19 of 63)	8.2% (22 of 269)	0% in prophylactic group 3.2% (3/95) in preemptive group
Cumulative incidence rate of HBV reactivation	10.4/100 person-year	41.5%/2 years	8.3%/1.5 years	N/A
Hepatitis	6.7% (10 of 150)	0%	0%	0% in prophylactic group 1.1% (1/95) in preemptive group
HBV reactivation related death	0%	0%	0%	0%

Abbreviations: R-CHOP, rituximab, cyclophosphamide, doxorubicin, vincristine and prednisolone.

**Table 4:** HBV reactivation in MM patients (case reports).

Reference	Age, Gender	MM Subtype	Treatment	Hepatitis	Antiviral Agents	Time of Initiating Antiviral Therapy	Outcome
Tapan (2011) [47]	64, male	IgG-κ	RT, DEX, TD, Bor with tanespimycin, Ld, CD, Benda with DEX	(+)	ETV	After hepatitis	Died of fulminant hepatic failure after 1 month of entecavir treatment
Tanaka (2012) [48]	72, male	IgG-κ	MPT, BD	(−)	ETV	Preemptive therapy	Alive with MM
Goldberg (2013) [49]	72, male	N/A	Thal, Len, Bor	(+)	ETV	After hepatitis	Died of hepatic failure
Hussain (2014) [50]	73, female	N/A	Vertebral decompression, RT, Bor and liposomal doxorubicin, Len	(−)	TDF	Preemptive therapy	Died of MM
Yang (2014) [51]	56, male	IgG-κ	CDEP, CVAD, Mel, ASCT, VTD-PACE, BLD	(+)	TDF	After hepatitis	Died of PH and bacteremia
	77, male	IgG-κ	DT-PACE, Mel, ASCT, IFN	(+)	LAM → switched to ETV	After hepatitis	Alive with MM (remission)
Silva-Pinto (2015) [52]	57, male	IgG-λ	TD, RT, ASCT, BD, Thal maintenance	N/A	ETV → added to TDF	N/A	Died of MM
Gu (2015) [53]	55, male	IgG-κ	VAD, ASCT, PSL maintenance	(+)	ETV	After hepatitis	Alive with MM
Danhof (2015) [54]	59, male	IgG-κ	VAD, tandem ASCT, Bor, Benda and Ld, AUY-922 and BD, BLCd, Pd	N/A	ETV	N/A	Died of septicemia
Almaghrabi (2017) [55]	68, male	N/A	BCD, ASCT, Len maintenance	(+)	ETV	After hepatitis	Alive

Abbreviations: ASCT, autologous stem cell transplantation; AUY-922, Hsp-90 inhibitor; BD, bortezomib and dexamethasone; BCD, bortezomib, cyclophosphamide and dexamethasone; Benda, bendamustine; BLCd, bortezomib, lenalidomide, cyclophosphamide and dexamethasone; BLD, bortezomib, lenalidomide, and dexamethasone; Bor, bortezomib; CD, cyclophosphamide and dexamethasone; CDEP, cyclophosphamide, dexamethasone, etoposide, and cisplatin; CVAD, cyclophosphamide, vincristine, doxorubicin, and prednisolone; DEX, dexamethasone; DT-PACE, dexamethasone, thalidomide, cisplatin, doxorubicin, cyclophosphamide, and etoposide; ETV, entecavir; IFN, interferon; LAM, lamivudine, Ld, lenalidomide and dexamethasone; Len, lenalidomide; Mel, melphalan; MM, multiple myeloma; MPT, melphalan, prednisolone, and thalidomide; Pd, pomalidomide and dexamethasone; PH, pulmonary hypertension; RT, radiotherapy; TD, thalidomide and dexamethasone; TDF, tenofovir; Thal, thalidomide; VAD, vincristine, doxorubicin, and dexamethasone; VTD-PACE, bortezomib, thalidomide, dexamethasone, cisplatin, doxorubicin, cyclophosphamide, and etoposide.

**Table 5:** HBV reactivation in MM patients (case series).

Reference	Number of Patients (MM Patients)	Number of Patients with HBV Reactivation (MM Patients)	Definition of HBV Reactivation	Number of Patients Who Developed Hepatitis (MM Cases)	Antiviral Agents	Number of Patients Who Died of Hepatitis (MM Patients)	Risk Factors
Endo (2000) [56]	47 (13)	3 (3)	Reappearance of HBsAg	3 (3)	Not received	0	Steroid
Uhm (2007) [57]	141 (53)	7 (6)	Reappearance of HBsAg	5 (N/A)	LAM	0	N/A
Matsue (2009) [58]	81 (12)	6 (1)	Reappearance of HBsAg	4 (0)	LAM, ETV	0	N/A
Ceneli (2010) [59]	90 (46)	3 (3)	Reappearance of HBsAg with increase in HBV DNA level	3 (3)	LAM	0	N/A
Yoshida (2010) [60]	15 (15)	2 (2)	HBV DNA level becomes detectable	1 (1)	ETV	0	N/A
Borentain (2010) [61]	84 (N/A)	7 (1)	HBV DNA level becomes detectable	7 (1)	LAM	3 (1)	>1 line of chemotherapy
Lee (2015) [62]	230 (230)	12 (12)	Reappearance of HBsAg	8	LAM, ETV, TDF	0	ASCT, anti-HBs negative
Li (2015) [63]	112 (112)	2 (2)	Loss of anti-HBs and reappearance of HBsAg Increase of HBV DNA level	N/A	LAM, ETV	0	N/A
Takahashi (2015) [8]	N/A	11 (4)	Reappearance of HBsAg or > 10-fold increase in HBV DNA or HBV-DNA level becomes detectable	4 (2)	LAM, ETV	4 (1)	N/A
Tsukune (2016) [64]	99 (99)	9 (9)	HBV DNA level becomes detectable	0	ETV	0	elevated serum albumin
Mochida (2016) [65]	289 (N/A)	20 (2)	HBV DNA level becomes detectable	N/A	ETV	N/A	N/A
Han (2016) [66]	738 (54)	23 (6)	Reappearance of HBsAg	N/A	ETV, TDF, LdT	1 (0)	loss of anti-HBs, ALL, MM
Varma (2017) [67]	107 (107)	7 (7)	Reappearance of HBsAgor > 10-fold increase in HBV DNA	N/A	LAM, TDF	0	N/A
Tsukune (2017) [22]	760 (760)	58 (58)	HBV DNA level becomes detectable	10	LAM, ETV	1	ASCT, lenalidomide *
Ataca Atilla P (2019) [68]	178 (178)	8 (8)	Loss of anti-HBs and reappearance of HBsAg Increase of HBV DNA level	N/A	LAM, TDF	N/A	N/A

* Lenalidomide significantly reduced risk of HBV reactivation. Abbreviations: ALL, acute lymphoblastic leukemia; anti-HBs, antibodies against hepatitis B surface antigen; ASCT, autologous stem cell transplantation; ETV, entecavir; LAM, lamivudine, LdT, telbivudine; MM, multiple myeloma; TDF, tenofovir.
